# PVA enema ameliorates DSS-induced acute colitis in mice

**DOI:** 10.1186/s12876-023-03005-w

**Published:** 2023-10-30

**Authors:** Zhiyong Zhang, Lingnan Kong, Ming Lv, Yukuan Yao, Li Gao, Ruchen Zhou, Wenlong Ma, Jing Li

**Affiliations:** 1 School of Clinical Medicine, Weifang Medical University, Weifang, China; 2 Department of Pathology, Zibo Central Hospital, 54 Gongqingtuan Xi Road, Zibo, 255036 Shandong China; 3 Zibo Central Hospital, Zibo, China; 4 Department of Outpatient, Zibo Central Hospital, Zibo, China; 5 Department of Gastroenterology, Qilu Hospital, Cheeloo College of Medicine, Shandong University, Jinan, China

**Keywords:** Colonic mucosal epithelium, Enema therapy, Organoids, Polyvinyl alcohol, Ulcerative Colitis

## Abstract

**Background:**

Ulcerative colitis (UC) represents a clinically challenging condition characterized by persistent damage to the colonic epithelial mucosa as the principal pathological feature. Polyvinyl alcohol (PVA) solution, primarily composed of glue, is a biodegradable polymer material that has found utility in the medical field. This research endeavors to investigate the therapeutic potential of PVA water solution in ameliorating UC in mice.

**Methods:**

UC was induced in 48 C57BL/6 mice by administering 2.5% DSS in their diet for 6 days. Mice were treated with different concentrations of PVA (0.1 mg/ml PVA, 0.3 mg/ml PVA, 1 mg/ml PVA, 3 mg/ml PVA, 10 mg/ml PVA) enemas (n = 6). Disease Activity Index (DAI) and histologic score were evaluated for inflammation degree. Furthermore, mouse colon organoids were cultured, which were used to assess the effects of PVA on expansion in vitro.

**Results:**

PVA aqueous solutions (1 mg/ml and 3 mg/ml) were able to alleviate the DAI in mice. By DAY 6, there was a significant 3/5-fold decrease in DAI within the 1 mg/ml PVA group (*p* = 0.02). Histopathology scores demonstrated improvements, while the levels of inflammatory factors in the intestinal mucosal tissue were reduced. Additionally, it was confirmed that PVA could promote the expansion of colonic organoids in vitro.

**Conclusions:**

In summary, our investigation has yielded findings indicating that PVA holds the potential to ameliorate symptoms associated with colitis in murine subjects afflicted by DSS-induced colitis, primarily through its facilitation of intestinal stem cell expansion. This study might provide a new candidate for the clinical treatment of ulcerative colitis.

**Supplementary Information:**

The online version contains supplementary material available at 10.1186/s12876-023-03005-w.

## Introduction

Polyvinyl alcohol (PVA) is a water-soluble polyhydroxy polymer that’s commonly used in regular glue production. However, due to the rise of biomaterial commercialization, PVA is gradually being applied to biomedical field [1]. PVA can serve as a coating agent for medications and dietary supplements [2], and it holds the potential to facilitate wound healing when combined with drugs [3]. By forming composite materials with other substances, PVA can be utilized as wound dressing to help reduce inflammation [4]. Furthermore, recent research indicates that PVA can contribute to creating nanofiber scaffolds, showing promise in promoting bone regeneration [5]. Researchers from the United States and Japan have discovered that substituting human serum albumin with PVA enables the expansion and stabilization of a large number of hematopoietic stem cells [6]. However, the impact of PVA on UC is not clear. This experiment is designed to observe how PVA affects a mouse model of UC and to determine the most effective concentration of PVA for therapeutic purposes.

## Materials and methods

### Preparation of solution

Preparation of Dextran sulfate sodium (DSS) solution: Distilled water was used to dissolve quantitative DSS (MP Biomedicals, Santa Ana, CA, USA), by stirring it on a magnetic agitator until fully dissolved. The solution was then diluted to a 2.5% DSS concentration using a measuring cup and stored at 4 °C. The molecular weight of DSS is 36,000–50,000 Da. For the PVA powder (Sigma-Aldrich, #363,146), it was dissolved in distilled water by heating and stirring on a heat agitator until complete dissolution. Further, the mixture was placed in a 50 °C incubator for 10 min to ensure full dissolution. Finally, the solutions were preserved at 4 °C.

### Animals and experimental design

**Fig. 1:**
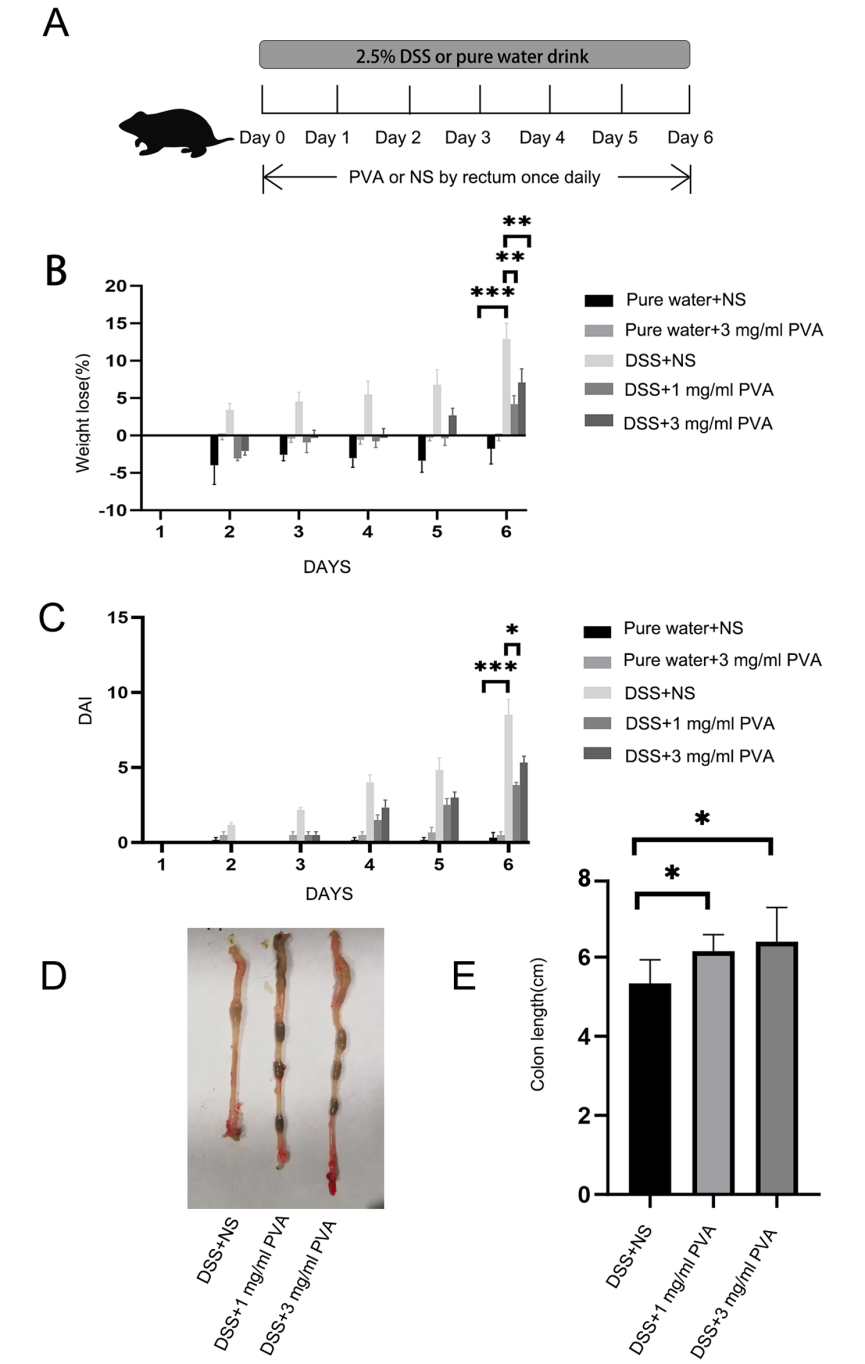
**Assessment of the effects of DSS treatment and PVA enema on mice**. (**A**) Schematic representation of the DSS treatment procedure coupled with PVA enema administration. (**B**) Variations in weight among five distinct mouse groups. (**C**) Disease activity index across the five mouse groups. (**D**) representation of Measurements of colonic length in mice from the groups: DSS + NS, DSS + 1 mg/ml PVA, and DSS + 3 mg/ml PVA. (**E**) The bar graph displays the colonic length for three groups: DSS + NS, DSS + 1 mg/ml PVA, and DSS + 3 mg/ml PVA. Each group is composed of 6 mice (N = 6). *p < 0.05, **p < 0.01, ***p < 0.001

C57BL/6 male mice of 6–8 weeks, weighing 22–26 g (Beijing HFK Biotechnology Co., Ltd.) were housed. The room temperature is controlled at 23–25 °C and a well-ventilated environment. Mice were randomly divided into 9 groups: (1) mice are fed with pure water daily and undergo normal saline (NS) enema once daily (pure water + NS), (2) with pure water daily and undergo 0.3 mg/ml PVA enema once daily (pure water + 0.3 mg/ml PVA), (3) with pure water daily and undergo 3 mg/ml PVA enema once daily (pure water + 3 mg/ml PVA), (4) with 2.5% DSS solution daily and undergo NS enema once daily (DSS + NS), (5) with 2.5% DSS solution daily and undergo 0.1 mg/ml PVA enema once daily (DSS + 0.1 mg/ml PVA), (6) with 2.5% DSS solution daily and undergo 0.3 mg/ml PVA enema once daily (DSS + 0.3 mg/ml PVA), (7) with 2.5% DSS solution daily and undergo 1 mg/ml PVA enema once daily (DSS + 1 mg/ml PVA), (8) with 2.5% DSS solution daily and undergo 3 mg/ml PVA enema once daily (DSS + 3 mg/ml PVA), (9) with 2.5% DSS solution daily and undergo 10 mg/ml PVA enema once daily (DSS + 10 mg/ml PVA). Each group of mice was raised in different cages, and each mouse wore ear labels to distinguish. To observe disease severity, clinical Disease Activity Index (DAI) is calculated daily. DAI includes the degree of body weight loss, stool consistency, and fecal blood. The total score is the sum of an individual grade of the above three indicators. For weight loss: 0 = no change, 1 = < 5%, 2 = 6–10%, 3 = 11–20%, 4 = > 20%. For stool: 0 = normal, 1 = soft and well-formed, 2 = soft without pellets, 4 = diarrhea. For blood: 0 = no blood, 1 = visible blood in rectum, 2 = grossing bleeding in rectum, 4 = visible blood on fur [7, 8]. mice were treated with DSS daily and the control group drinks pure water. Mice were given daily PVA or NS enemas. Furthermore, mice were not restricted from drinking the DSS solution, PVA was administered as an enema once a day at the fixed time (between 20:00 and 21:00) (Fig. 1A). The mice were given enemas using a disposable central venous catheter (Foshan Special Medical Co., Ltd) with 0.2-0.3ml PVA solution. Each mouse was placed in a separate cage to wait for defecation after enema treatment every day, and then the fecal status was observed. Fecal occult blood kit (dry chemical) (Ameritech Diagnostic Reagent (Jiaxing) Co., Ltd.) was used to determine fecal occult blood in mice. The principle is that hemosiderin in hemoglobin catalyzes hydrogen peroxide, and then releases new ecological oxygen, and the oxidized guaiacol turns blue.

### Histology

Mice are sacrificed using cervical dislocation. Dissect the mice on the day of sacrifice. The length of the colon was measured, and the lesion was clearly placed in 10% neutral formalin. After dehydration, it was embedded to make paraffin, and 4 mm sections were stained with hematoxylin and eosin. Pathological sections were scanned using a digital pathology slide scanner (NINGBO KONFOONG BIOTECH INTERNATIONAL CO., LTD). The histological damage score consists of four aspects, (1) severity of inflammation: no or rare inflammatory cells in lamina propria (LP) = 0, increased inflammatory cells in LP = 1, extension of inflammatory cells to the submucosa = 2, downward infiltration of inflammatory cells through the LP = 3; (2) depth of damage: no mucosal damage = 0, epithelial mucosal discrete lesions = 1, mucosal erosions or ulcers = 2, lesions breaking through the lamina propria downward infiltration = 3; (3) Injury of crypt: no damage = 0, less than 5% affected = 1, less than 33% damage = 2, less than 66% damage = 3, complete disappearance of crypt and epithelium = 4, (4) the percentage of the area involved: 1–25% = 1, 26–50% = 2, 51–75% = 3, 76–100% = 4. The total score is the sum of an individual’s grades [9, 10]. Histological assessment involved the meticulous examination of 10 random high-power field views (magnification of 20×) on each slide. This assessment was conducted in a double-blind manner, involving the interpretation of results by two independent pathologists to prevent bias.

### Detection of inflammatory cells and immunohistochemistry

Paraffin Sect. (3 mm thick) were for immunohistochemical staining. CD45 (murine monoclonal, 2B11&PD7/26; Guangzhou LBP Medicine Science & Technology Co., Ltd., China), MPO (Rabbit Polyclonal; Guangzhou LBP Medicine Science& Technology Co., Ltd., China), CD68 (murine monoclonal, LBP1-CD68; Guangzhou LBP Medicine Science & Technology Co., Ltd., China) are used to detect inflammatory factors levels. Ki-67 (murine monoclonal, MIB-1; Guangzhou LBP Medicine Science & Technology Co., Ltd., China) is used to detect cell proliferation. The immunohistochemical score was semi-quantitative. The score was based on the percentage of positive cells in the total cells (positive percentage). When the staining strength is uneven, the score is based on the maximum strength. The total score is obtained by multiplying the positive percentage score by the staining intensity score. The positive percentage was scored as follows: the range of less than 10% = 0, the staining area of 10–50% = 1, the staining area of 50–75% = 2, the staining area of more than 75% = 3. The staining strength was scored as follows: negative staining = 0, weak staining = 1, medium staining = 2, strong staining = 3 [11, 12]. The method for immunohistochemical scoring remained consistent with that utilized for histological scoring. Positive staining for CD45 occurred at the cell membrane, CD68 and MPO positive staining occurred in the cytoplasm, and Ki-67 positive staining was observed in the nucleus.

### Colonic crypt isolation and culture

Wild type mouse (C57BL/6, 6–8 weeks) was sacrificed. As before, cervical dislocation was used. Colon was flushed with cold PBS to remove the colon contents, opened longitudinally and chopped into around 5 mm pieces. The tissue was washed 3–5 times with cold PBS to remove fecal residue and bacteria, and further incubated in 2 mM EDTA with PBS for 60 min on ice. After removing EDTA, the tissue fragments were blown using a 5 ml pipette with cold PBS. The supernatant was collected and centrifuged at 300 g for 5 min at 4 °C. The sediment was resuspended with 1 ml DMEM, and the supernatant was enriched for crypts. 20 µl suspension was taken to microscopic examination and a fraction of supernatant was taken to 15 ml centrifuge tube that ensured 200–500 crypts/50 µl Matrigel (Corning®, #356,231). The supernatant was centrifuged at 200 g for 3 min at 4 °C and single cells were removed. The sediment was resuspended with cold DMEM, and mixed with an equal volume of melted Matrigel. Matrigel was seeded into 48-well culture plates (40 µl per well) prewarmed at 37 °C, and transferred into the incubator for 20 min. After Matrigel solidification, 250 µl per well Mouse Colonic Organoid Expansion Medium (biogeneous™, #K2204-MC) were added to 48-well culture plates. One day later, crypts were stimulated with PVA (Sigma-Aldrich, #363,146) 0, 1, 3 mg/ml. The medium was replaced every 3 days. On day 6, Cell Recovery Solution (Corning, #354,253) was added to the wells to solubilize Matrigel and collect organoids.µl.

### Statistical analysis

**Fig. 2:**
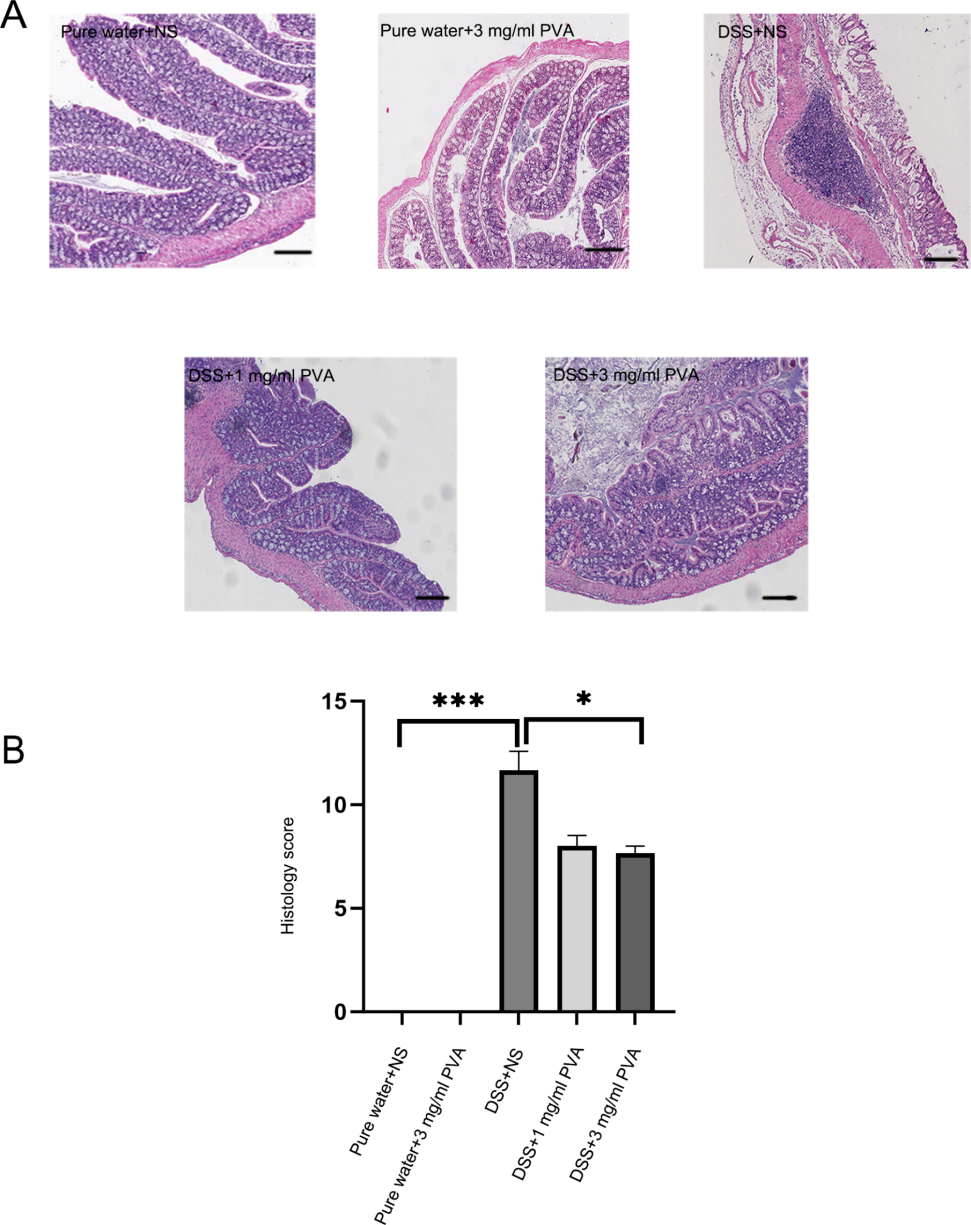
**Evaluation of PVA’s Impact on Colonic Epithelium**. (**A**) Representative sections from five distinct colonic tissue groups visualized using H&E staining; scale set at 100 μm. (**B**) The bar graph delineates the histopathological assessment scores for the colonic tissues across five mouse cohorts. Each cohort comprises 6 subjects (N = 6). Significance levels are indicated as follows: *p < 0.05, **p < 0.01, ***p < 0.001

The data were presented as mean values, with error bars indicated Standard Error of Mean (SEM). Computations and charting were performed using GraphPad Prism 8. Upon identifying significant effects via one-way ANOVA tests, detailed pairwise comparisons were discerned through Bonferroni *post hoc* analysis. For data that didn’t adhere to normal distribution, as observed in Fig. 2B, the Kruskal-Wallis Test was employed, followed by individual comparisons using Dunn test for statistical significance evaluation. The significance level was set as *p* < 0.05.

## Results

### PVA improved clinical symptoms in mice

Our investigation unveiled that the employment of a PVA aqueous solution led to a reduction in weight loss in mice subjected to DSS-induced conditions, consequently resulting in a decrease in DAI. By the sixth day, the DSS + NS group displayed a more pronounced extent of weight loss when juxtaposed with the pure water group (*p* < 0.001). Conversely, the 1 mg/ml PVA group exhibited a mitigated rate of weight loss in comparison to the DSS + NS group (*p* = 0.001), with weight loss also exhibiting a decrease within the 3 mg/ml PVA group (*p* = 0.009) (Fig. 1B: one-way ANOVA comparing the five groups; F _(4,25)_ = 16.154; *p* = 0.000001; n = 6). In contrast to the pure water group, the DAI observed within the DSS + NS group manifested a marked increase (*p* < 0.001). Yet, a notable decrease in DAI was noted within the 1 mg/ml PVA group (*p* = 0.02), when contrasted with the model group (Fig. 1C: Kruskal-Wallis; *p* = 0.00001; n = 6). Furthermore, comparative assessment demonstrated an augmentation in colon length within both the 1 mg/ml PVA (*p* = 0.04) and 3 mg/ml PVA groups (*p* = 0.03), as opposed to the model group (Fig. 1D–E: one-way ANOVA comparing the three groups; F _(2,15)_ = 5.349; *p* = 0.01; n = 6). Of additional note, it was observed that the utilization of PVA at concentrations of 0.1 mg/ml, 0.3 mg/ml, and 10 mg/ml did not yield statistically significant reductions in terms of weight loss and DAI when juxtaposed with the DSS group (Supplementary Fig. 1A–B).

### PVA improves colonic mucosal epithelial injury in mice

To explore the impact of an aqueous solution of PVA on injury to the epithelial lining of the colonic mucosa, an assessment of histopathological scores was conducted across distinct experimental cohorts. As depicted in Fig. 2A-B, discernible alterations were observed in the architecture of the intestinal mucosa within the DSS + NS group, characterized by a conspicuous diminishment in crypt structures (*p* < 0.001). Conversely, administration of PVA exhibited a discernible amelioration in mucosal impairment. The application of PVA at concentrations of 3 mg/ml (*p* = 0.04) yielded noteworthy reductions in pathological scores relative to the DSS group (Fig. 2B: Kruskal-Wallis; *p* = 0.00002; n = 6). However, among the cohorts subjected to alternate concentrations of PVA, namely 0.1 mg/ml, 0.3 mg/ml, or 10 mg/ml, discernable attenuation of mucosal epithelial injury was not evidenced (Supplementary Fig. 2).

### PVA reduces levels of inflammatory factors in the colon of mice

**Fig. 3:**
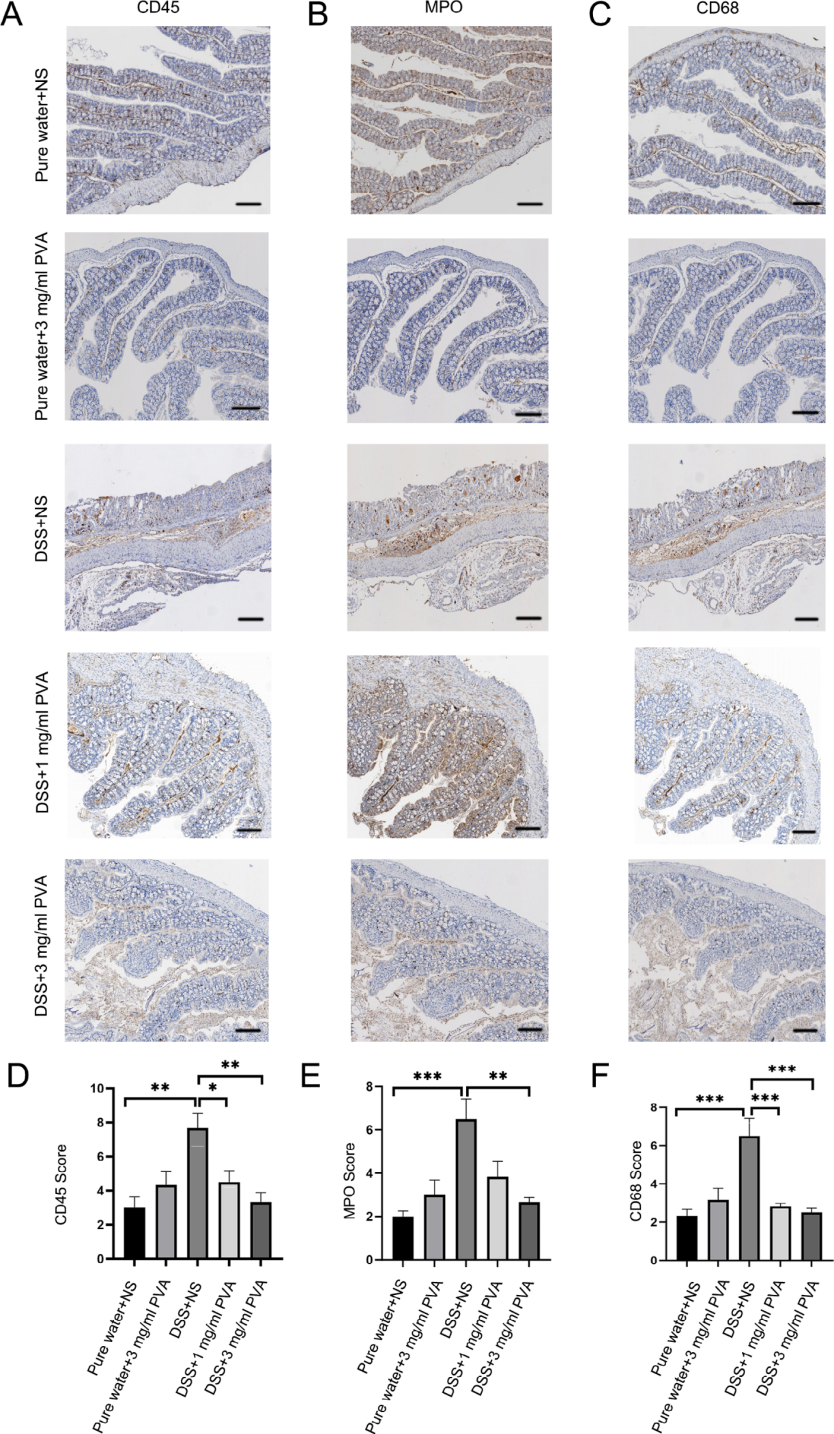
**PVA’s Influence on Colonic Inflammation**. (**A**-**C**) Demonstrative immunohistochemical profiles of CD-45 (characterized by cell membrane staining), MPO, and CD68 (both displaying cytoplasmic staining) within the mouse colonic mucosa. A marked increase in leukocyte infiltration (highlighted in brown) is observed in the distal colon mucosa of the DSS + NS group, which is mitigated upon PVA treatment; visual reference scale is set at 100 μm. (**D**-**F**) Bar charts present a comparative evaluation of inflammatory intensity across the studied cohorts. Each group encompasses 6 mice (N = 6). Significance annotations: *p < 0.05, **p < 0.01, ***p < 0.001

We conducted an assessment of inflammation using immunohistochemical sections in order to observe the presence of inflammatory cells within a murine model of colitis. In comparison to the control group receiving pure water, the cohort exposed to DSS exhibited a notably heightened extent of leukocyte infiltration within the distal colonic mucosal layer (Fig. 3A-C). Compared with the control group, the DSS + NS group had higher CD45 (*p* = 0.001), MPO (*p* < 0.001), and CD68 (*p* < 0.001) scores (Fig. 3D-F). Notably, when compared to the DSS-exposed group, there was a statistically significant reduction in immunohistochemical CD45 score observed within both the 1 mg/ml PVA-treated group (*p* = 0.03) and the 3 mg/ml PVA-treated group (*p = 0.001*) (Fig. 3D: one-way ANOVA comparing the five groups; F _(4,25)_ = 8.046; *p* = 0.0002; n = 6). Similar reductions were observed in the immunohistochemical myeloperoxidase (MPO) score within the 3 mg/ml PVA-treated group (*p* = 0.002) when compared to the DSS + NS group. There was a downward trend in the 1 mg/ml PVA-treated group (*p* = 0.05) (Fig. 3E: one-way ANOVA comparing the five groups; F _(4,25)_ = 7.970; *p* = 0.0002; n = 6). Furthermore, the immunohistochemical CD68 score was found to be significantly diminished in both the 1 mg/ml PVA-treated group (*p* < 0.001) and the 3 mg/ml PVA-treated group (*p* < 0.001) in comparison to the DSS + NS group (Fig. 3F: one-way ANOVA comparing the five groups; F _(4,25)_ = 10.635; *p* = 0.00003; n = 6). Notably, the levels of inflammatory factors remained unchanged following treatment with 0.1 mg/ml PVA, 0.3 mg/ml PVA, or 10 mg/ml PVA, as depicted in Supplementary Fig. 3.

### PVA promotes colonic epithelial cell and colon organoids proliferation

**Fig. 4:**
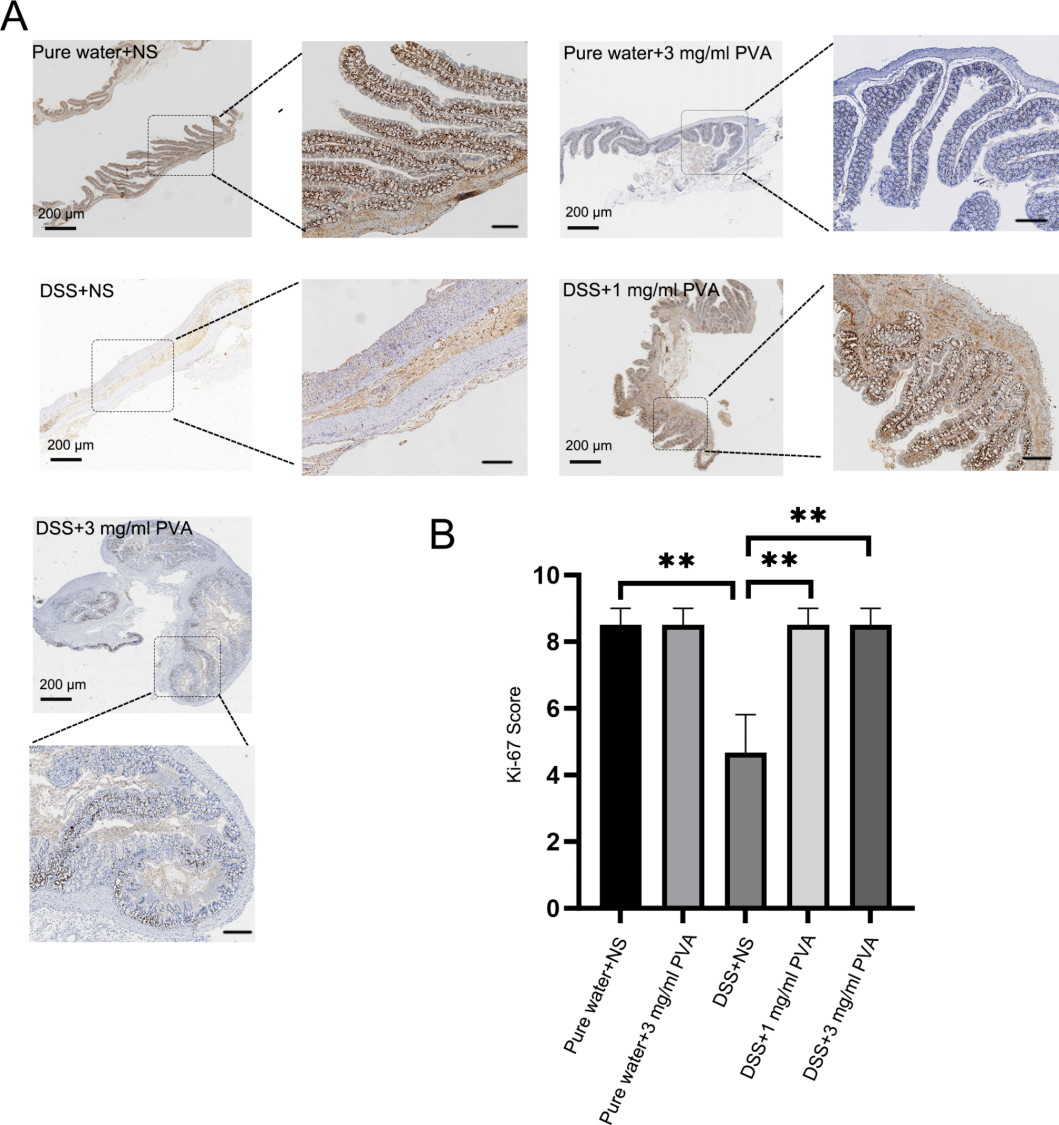
**PVA’s Impact on Colonic Epithelial Cell Proliferation in Mice**. (**A**) Demonstrative immunohistochemical representation of ki-67 (identified by nuclear staining) in the colon mucosa across the distinct groups; reference scale set at 100 μm, reference scale of the zoomed image set at 200 μm. (**B**) Bar charts illustrate the ki-67 scores among the various cohorts. Each cohort consists of 6 mice (N = 6). Significance indicators: **p < 0.01

**Fig. 5:**
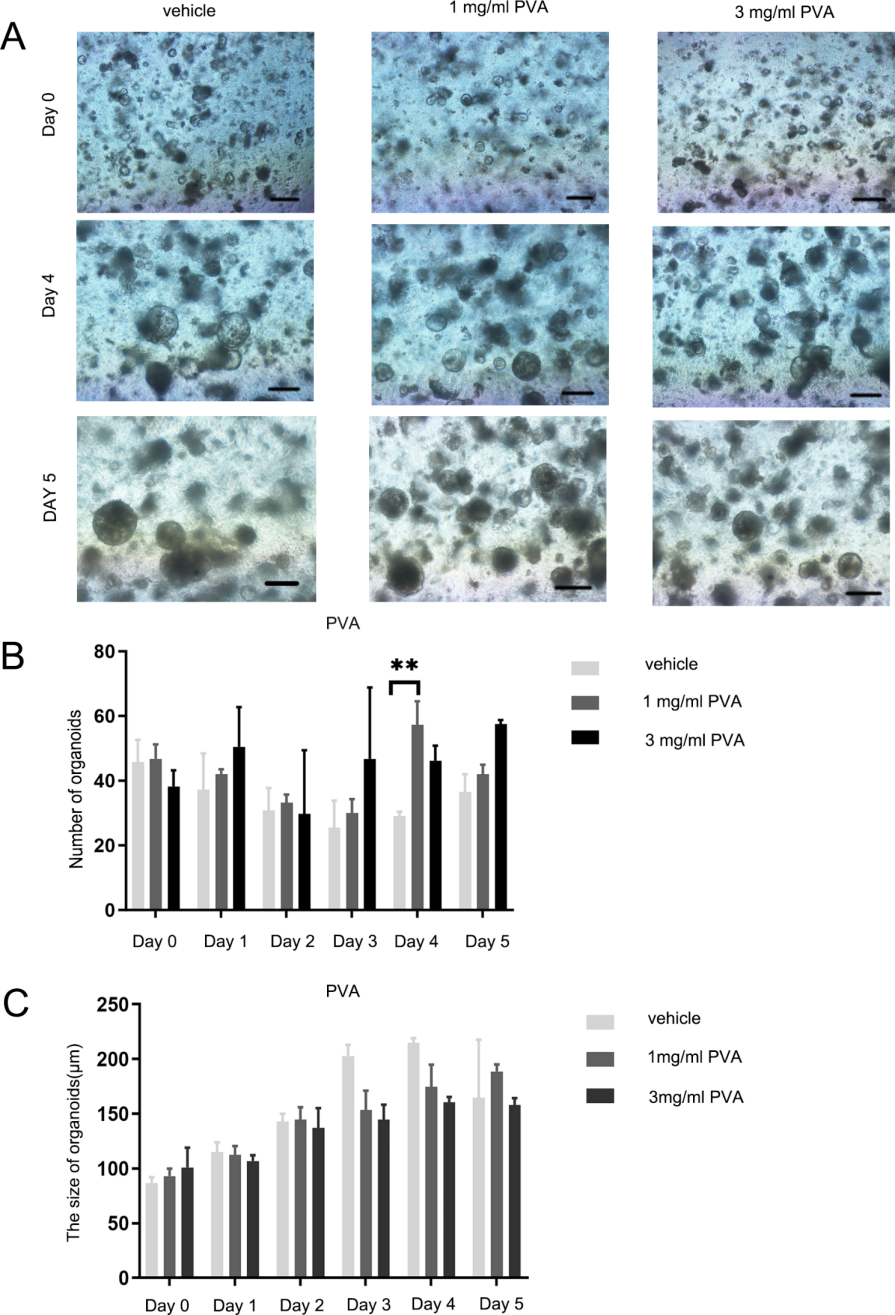
**PVA’s Influence on Mouse Colon Organoids**. (**A**) Exemplary visuals illustrating the impact of varying PVA concentrations on organoids over a 5-day period, with a reference scale of 100 μm. (**B**) Bar graphs present the count of organoids across distinct cohorts. (**C**) Bar graphs detail the dimensional assessment of organoids among the various groups. Significance levels are indicated as: **p < 0.01

To ascertain the impact of PVA on the proliferation of murine colonic epithelial cells, we evaluated the immunohistochemical expression of Ki-67. A notable reduction in mouse epithelial cell proliferation was observed in the group exposed to DSS as compared to the control group receiving pure water (*p* = 0.005). Contrastingly, the immunohistochemical Ki-67 scores were significantly elevated in the presence of 1 mg/ml PVA (*p* = 0.005) and 3 mg/ml PVA (*p* = 0.005) when juxtaposed with the DSS-treated group (Fig. 4A–B: one-way ANOVA comparing the five groups; F _(4,25)_ = 6.358; *p* = 0.001; n = 6). Conversely, the Ki-67 score exhibited no significant alteration within the 0.1 mg/ml PVA, 0.3 mg/ml PVA, or 10 mg/ml PVA groups, as illustrated in Supplementary Fig. 4. Furthermore, through in vitro cultures of murine colon organoids, it was evident that the inclusion of PVA yielded discernible effects. Specifically, the treatment with 1 mg/ml PVA led to a notable increase in the number of colonic organoids. By the fourth day of culture, the 1 mg/ml PVA (*p* = 0.004) group exhibited elevated organoid counts in comparison to the vehicle group (Fig. 5B: one-way ANOVA comparing the three groups; F _(2,9)_ = 10.318; *p* = 0.005; n = 4). Additionally, it did not show that a particular effect of PVA on size of organoid. (Fig. 5A, C).

## Discussion

In this study, it was ascertained that PVA demonstrates efficacy in mitigating tissue damage, ameliorating weight loss, attenuating colonic epithelial inflammatory cell count, and reducing DAI in a murine model of colitis induced by DSS. PVA, distinguished for its non-toxic nature, underscores advantageous attributes encompassing favorable biocompatibility and robust stability. This polymer finds application across diverse domains, notably including its incorporation as a carrier for pharmaceutical agents to facilitate wound healing. A pertinent literature citation posits the utilization of curcumin, administered orally, in the treatment of UC, in conjunction with PVA and guar gum. The amalgamation of PVA and guar gum yields gel matrices characterized by commendable biocompatibility, enabling efficacious encapsulation and systemic deployment of curcumin, thereby contributing to physiological effects [13]. However, the therapeutic implications of PVA have heretofore remained unexplored. Our investigation underscores the potential of PVA in ameliorating symptoms and inflammation associated with UC.

UC is an inflammatory bowel disease that can induce inflammation and ulcers in the digestive tract. It is prevalent worldwide due to diet and environmental factors [14]. The clinical features include diarrhea, abdominal pain, weight loss and bloody stools, which could seriously affect patients’ normal work and greatly reduce the quality of life [15]. A retrospective study shows that UC patients have a higher risk of experiencing intestinal complications (bleeding, perforation, abscess, etc.) and extraintestinal manifestations (iron-deficiency anemia, ankylosing spondylitis, vasculitis, etc.) than healthy people do in their lifetime [16]. In addition, UC can also lead to the development of colorectal cancer [17]. Furthermore, in recent years, the prevalence of UC in children is gradually increasing [18].

However, there hasn’t been a specific drug developed in recent years exclusively for treating UC. Although certain drugs like 5-aminosalicylic acid, glucocorticoids, methotrexate, tofacitinib, biological agents targeting anti-TNF, and anti-interleukin agents have shown some effectiveness, they come with varying degrees of side effects [19]. Despite the advancements in the modification of 5-aminosalicylic acid (5-ASA) and its subsequent administration, the noticeable adverse effects persist, including diarrhea and abdominal pain. In addition, patients who discontinue treatment with 5-ASA are at higher risk of adverse clinical outcomes, such as hospital admission [20]. Another study also indicates that the administration of 5-ASA fails to mitigate the likelihood of colon cancer occurrence [21]. Corticosteroids, treated for short-term therapeutic interventions, are beset by a multitude of complications upon extended usage, encompassing osteoporosis and hypertension [22, 23]. Previous case report revealed that, despite their therapeutic potential, the prolonged treatment of immunomodulatory agents might lead to the precancerous lesions such as high-grade squamous intraepithelial lesions [24]. While anti-TNF can be used for severe ulcerative colitis treatment, there’s a risk of recurrence if it is discontinued [25]. Furthermore, biologic agents, exemplified by anti-TNF drugs, introduce an elevated vulnerability to infections among UC patients, concurrently posing risks of diverse tuberculosis manifestations [26, 27]. Notwithstanding the reduction in surgical interventions post-biologic usage, the lingering disease susceptibility coupled with postoperative complications remains a persistent concern [28]. UC patients have more complications than normal patients undergoing surgery [29].

Insights gleaned from select investigations underscore that abating inflammatory cell populations holds promise in alleviating colonic epithelial impairments in murine models [30, 31]. Furthermore, antecedent reports propose a plausible correlation between epithelial apoptosis and mucosal barrier functionality. In instances of UC, augmented apoptotic cell and histiocyte counts within the lamina propria are observed, the latter facilitating phagocytosis of apoptotic cells [32]. Scholarly discourse highlights the pivotal role of intestinal epithelial cell demise in perpetuating colitis-associated inflammation and compromised barrier integrity [33, 34]. The confluence of increased intestinal epithelial cell mortality and heightened inflammatory cell presence precipitates the enduring course of UC. Our investigative inquiry culminates in the discernment that PVA effectively stimulates intestinal epithelial cell expansion. This emergent insight augments the premise that PVA may hold the potential to ameliorate mucosal damage via its facilitative effect on intestinal epithelial cell expansion.

The disruption of intestinal crypt cells stands as a contributory factor in the pathogenesis of afflictions. [35]. Stem cells, orchestrators of intestinal epithelial cell turnover, play a pivotal role in the modulation of inflammatory processes [36]. Emerging evidence underscores the potential linkage between the impairment of intestinal epithelial stem cells and the onset of colitis [37]. Endeavors to expand hematopoietic stem cells in vitro have hitherto met with limited success, with joint efforts by American and Japanese researchers elucidating human serum albumin’s inhibitory role in hematopoietic stem cell expansion. Subsequent investigations identified PVA as a suitable replacement for human serum albumin, thereby facilitating successful and sustained expansion of hematopoietic stem cell populations [6]. Noteworthy research has elucidated a close interrelation between the expansion of intestinal epithelial cells in murine models exposed to DSS and the recuperation of the intestinal mucosal lining. [38, 39]. Within the framework of our inquiry, the administration of PVA led to an augmented quantity of colon organoids in murine subjects. However, the precise underlying mechanism remains veiled. Our empirical findings indicate an elevation in the number of colon organoids derived from epithelial stem cells following PVA administration, thereby positing a potential mechanistic foundation for PVA’s facilitation of intestinal epithelial cell expansion. Notably, further rigorous investigation is imperative to corroborate these preliminary findings in subsequent studies.

## Conclusion

In summary, our investigation has yielded findings indicating that PVA holds the potential to ameliorate symptoms associated with colitis in murine subjects afflicted by DSS-induced colitis, primarily through its facilitation of intestinal stem cell expansion. Of notable significance is the economically viable nature of PVA coupled with its established safety profile. Consequently, this study introduces a novel and secure therapeutic avenue for addressing UC.

Abbreviation DSS - Dextran sulfate sodium; PVA - Polyvinyl alcohol.

### Electronic supplementary material

Below is the link to the electronic supplementary material.

Supplementary Material 1

## Data Availability

All data that support the findings of this study are available upon request from the corresponding author.

## Abbreviations

PVA: Polyvinyl alcohol
UC: Ulcerative colitis
DSS: Dextran sulfate sodium
NS: Normal saline
DAI: Disease activity index
PBS: Phosphate buffer saline
DMEM: Dulbecco’s Modified Eagle Medium
IHC: Immunohistochemical
SEM: Standard Error of the Mean
ANOVA: Analysis of Variance
