# Supplementary material for: PVA enema ameliorates DSS-induced acute colitis in mice
Source: BMC Gastroenterol. 2023 Oct 30;23:368. doi: 10.1186/s12876-023-03005-w (PMC10617076; doi:10.1186/s12876-023-03005-w)
Supplement: Supplementary file 1 — Supplementary Material 1 [file 12876_2023_3005_MOESM1_ESM.docx]

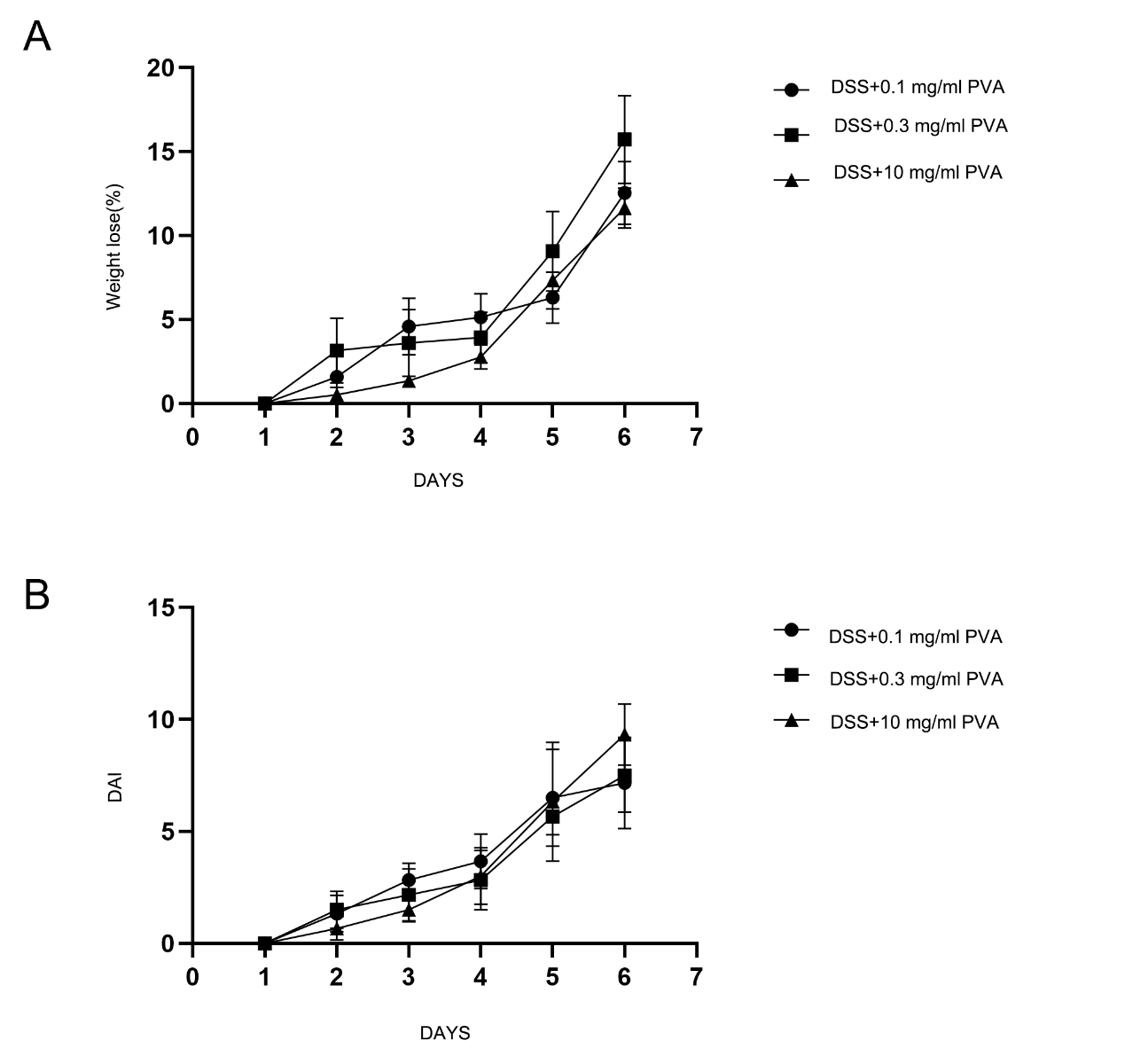


**Supplementary Fig1.** **Effects of PVA on clinical symptoms.**

(A) Weight changes of mice in 3 groups. (B) Disease activity index of 3 groups of mice. N = 6 per group. DSS, Dextran sulfate sodium. PVA, Polyvinyl alcohol.


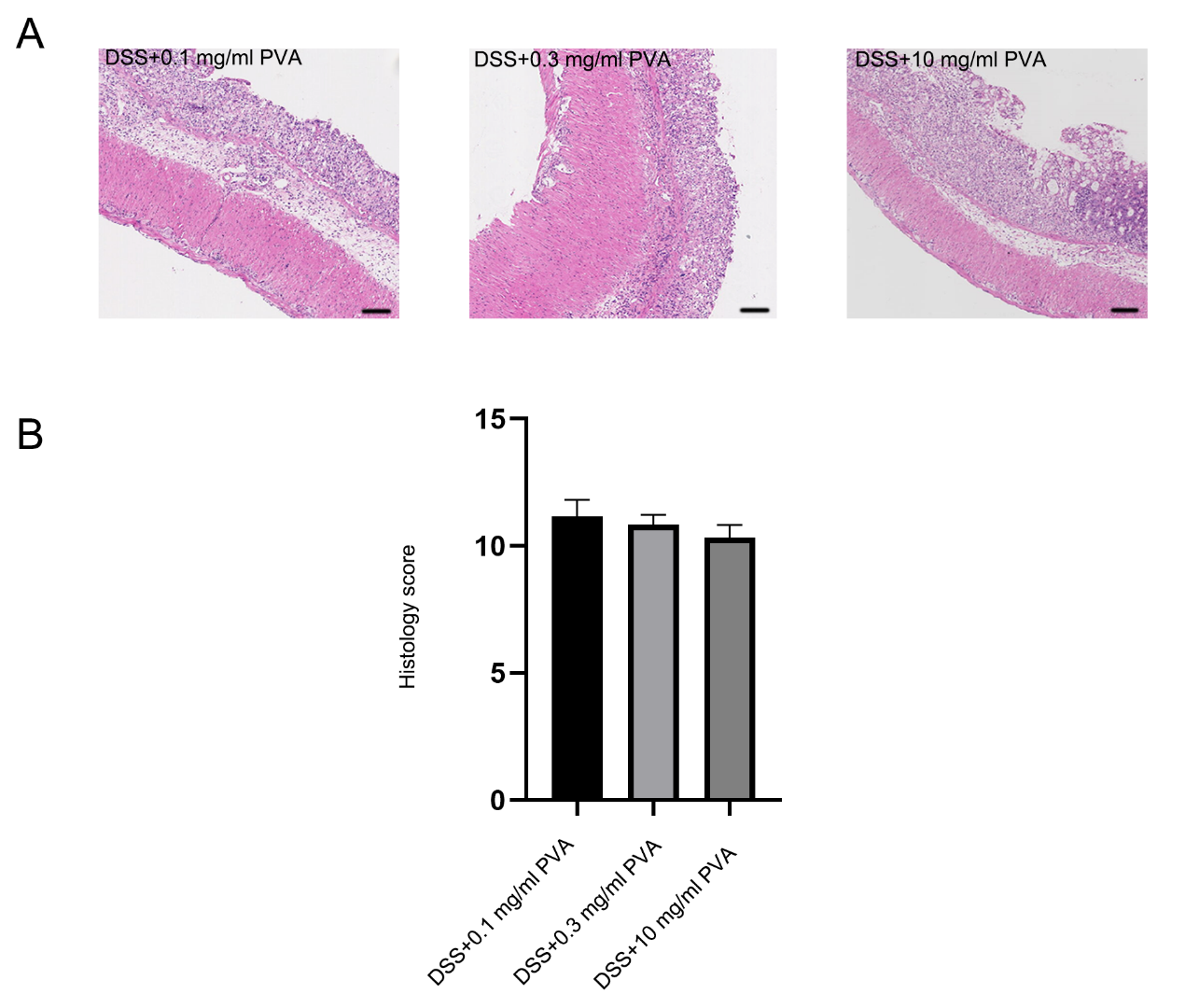


**Supplementary Fig2.** **Effects of PVA on the colonic epithelium of mice.**

(A) Representative fractions of 3 groups of colonic tissue stained by H&E, scale bar 100 µm. (B) Colonic histopathology scores in 3 groups of mice. N = 6 per group. DSS, Dextran sulfate sodium. PVA, Polyvinyl alcohol.


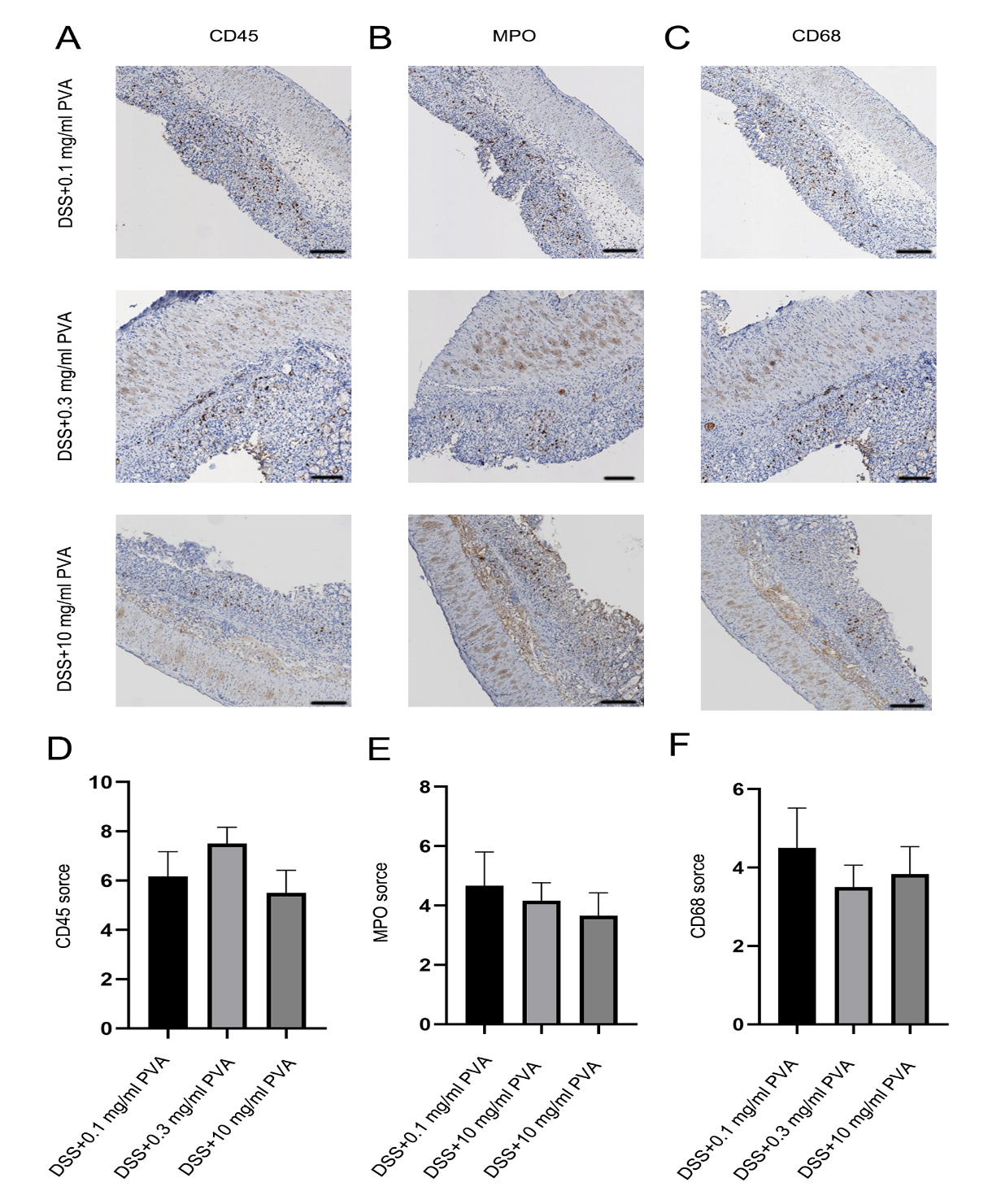


**Supplementary Fig3.** **Effects of PVA on colon inflammation.**

(A-C) Representative immunohistochemical data of CD-45, MPO, CD68 staining in mouse colonic mucosa, scale bar 100 µm. (D-F) Bar graphs depicting inflammatory severity within the different groups. N = 6 per group. DSS, Dextran sulfate sodium. PVA, Polyvinyl alcohol.


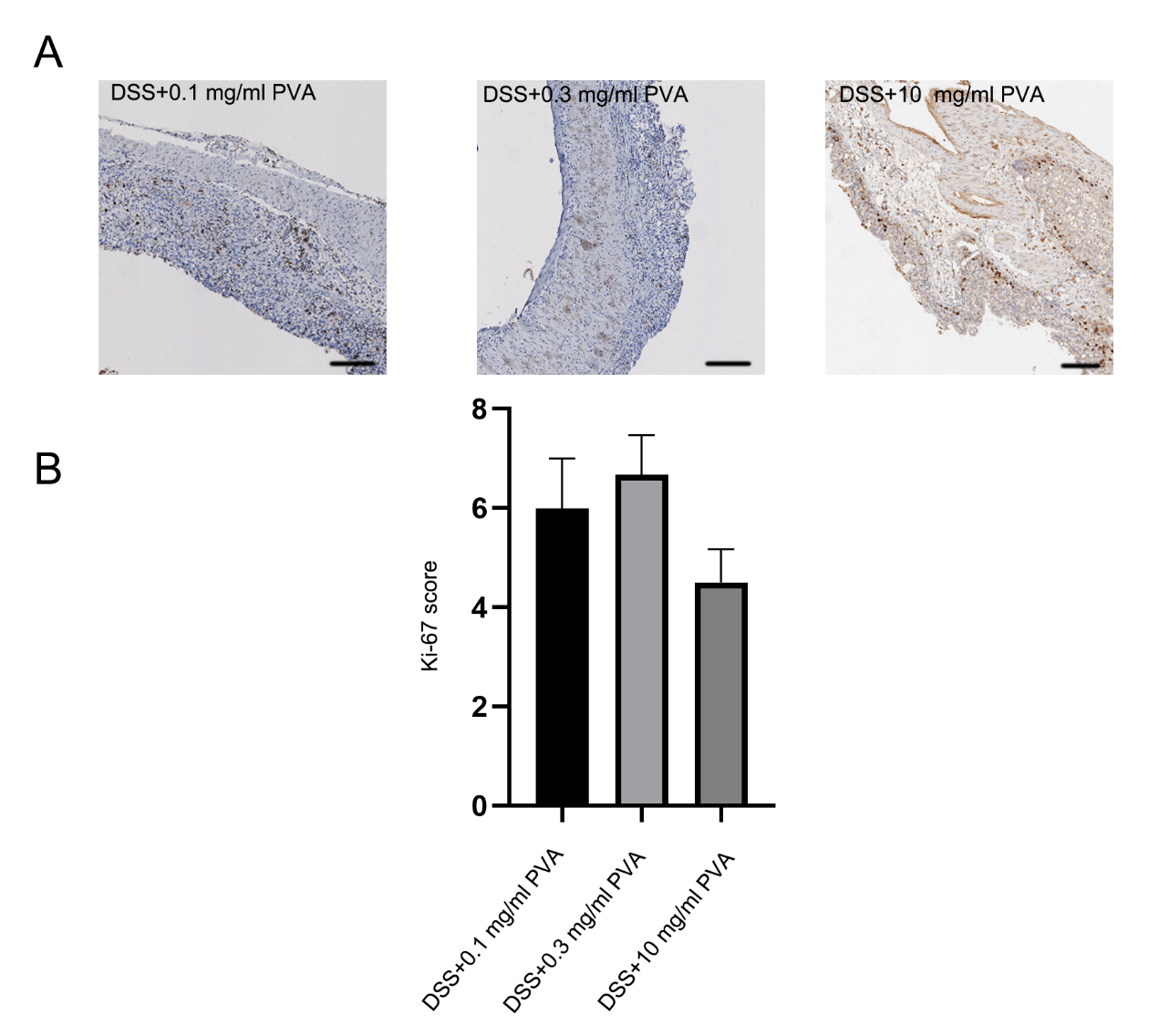


**Supplementary Fig4.** **Effects of PVA on proliferation of colonic epithelial cells in mice.**

(A) Representative immunohistochemical staining of ki-67 (brown) in colon mucosa within the different groups, scale bar 100 µm. (B) Bar graphs depicting ki-67 score in different groups. N = 6 per group. DSS, Dextran sulfate sodium. PVA, Polyvinyl alcohol.
